# Removal of Warts

**Published:** 1884-08

**Authors:** 


Removal of Warts.—Dr. Et. Guenot reports (*Bull. Gen. de Ther.*) that he has removed a large crop of warts, occurring on the hands of a patient, by giving daily a ten grain dose of calcined magnesia in the morning before breakfast.

